# Macrocyclizing-thioesterases in bacterial non-ribosomal peptide biosynthesis

**DOI:** 10.1007/s11418-024-01841-y

**Published:** 2024-08-30

**Authors:** Kenichi Matsuda

**Affiliations:** Faculty of Pharmaceutical Sciences, Hokkaido University, Kita 12, Nishi 6, Kita-ku, Sapporo, 060-0812 Japan

**Keywords:** Biosynthesis, Non-ribosomal peptide, Macrocyclization, Thioesterase, Chemoenzymatic synthesis

## Abstract

Macrocyclization of peptides reduces conformational flexibilities, potentially leading to improved drug-like properties. However, side reactions such as epimerization and oligomerization often pose synthetic challenges. Peptide-cyclizing biocatalysts in the biosynthesis of non-ribosomal peptides (NRPs) have remarkable potentials as chemoenzymatic tools to facilitate more straightforward access to complex macrocycles. This review highlights the biocatalytic potentials of NRP cyclases, especially those of cis-acting thioesterases, the most general cyclizing machinery in NRP biosynthesis. Growing insights into penicillin-binding protein-type thioesterases, a relatively new group of trans-acting thioesterases, are also summarized.

**Graphical abstract:**

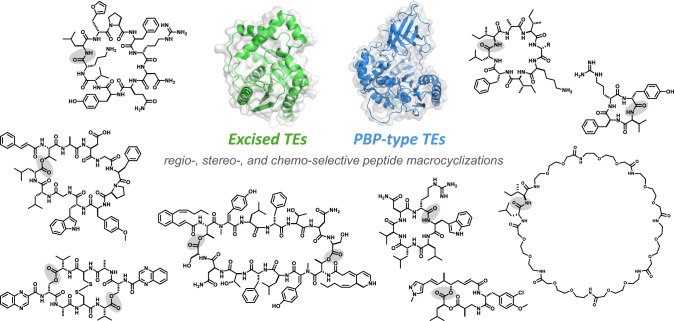

## Introduction

Cyclic peptides tend to exhibit improved metabolic stability, membrane permeability, and target specificity as compared to their linear counterparts [1–5]. Consequently, macrocyclization is a significant modification in the development of peptide-based therapeutics. However, despite its importance, efficient construction of macrocyclic scaffolds remains a challenging task, due to inherently competing side reactions like oligomerization and epimerization [6]. Under such circumstances, the use of biocatalysts has garnered keen attention due to their high selectivity and mild reaction conditions. Such peptide-cyclizing biocatalysts with synthetic potential have been identified mainly from secondary metabolism in bacteria and plants [7, 8].

Numerous pharmaceutically important macrocyclic peptides are biosynthesized through non-ribosomal pathways [9]. Representatives of clinically approved non-ribosomal peptides (NRPs) include the immunosuppressive agent cyclosporin, lipopeptide antibiotics like daptomycin and polymyxin, and glycopeptide antibiotics like vancomycin. NRPs are generally biosynthesized by non-ribosomal peptide synthetases (NRPSs). These megasynthetases consist of several functional domains that cooperatively assemble amino acid building blocks to produce grown peptide intermediates that are covalently bound to the synthetase. After assembly, peptide chains are processed by the C-terminal thioesterase domain (TE) and released from the NRPS as linear or cyclic products. Although several enzymatic machineries that mediate cyclization of NRPs have been identified, TEs are the most general biosynthetic mechanisms to generate macrocyclic scaffolds [10–12].

TE is a serine protease-type enzyme with a typical α/β hydrolase fold equipped with a Ser/His/Asp catalytic triad. TE-mediated macrocyclization proceeds via two steps. First, the grown linear peptide intermediate is transferred onto the catalytic Ser to form the peptide-*O*-TE complex. Subsequent attack from intramolecular nucleophiles on the ester carbonyl achieves the cyclization and release of the cyclic products from TEs, while attack from water results in the release of linear products. The hallmark of the TE-catalyzed cyclization is the diversity of nucleophiles used for cyclization: TEs utilize not only N-terminal *N*α-amine nucleophiles, but also other nucleophiles such as the β-OH of an N-terminal acyl group, *Nω*-amine of basic amino acids, β-OH of Ser/Thr/β-OH Phe, phenolic hydroxy of Tyr, and thiol of Cys, resulting in lariat-shaped macrocycles with various linkages at the ring closing site [10, 11]. Moreover, some TEs catalyze ligation along with cyclization, yielding cyclo-dimers and cyclo-trimers [10, 11]. As the functions of TEs have enormous impacts on the molecular shapes of the final products, TEs can be considered as major contributors toward generating the structure diversity of NRPs.

In 2000, Walsh and colleagues reconstituted the cyclization activity of TE excised from tyrocidine synthetase in vitro, and chemoenzymatically synthesized tyrocidine from a linear peptide using a phosphopantetheine surrogate: *N*-acetylcysteamine (SNAC) [13]. This work set the stage for the exploitation of excised TEs as chemoenzymatic tools for macrocyclization. Since then, TEs from various biosynthetic pathways have been characterized in vitro, with some of them successfully facilitating the chemoenzymatic synthesis to afford access to natural products and libraries of their analogs.

This review focuses on macrocyclizing-TEs in the biosynthesis of bacterial NRPs and their utilization as chemoenzymatic tools, with special emphasis on recent reports. This review also highlights the growing insights into penicillin-binding protein-type thioesterases (PBP-type TEs), a new group of NRP cyclases with remarkable biocatalytic potentials [14–16].

### TE-catalyzed head-to-tail macrocyclization

**Fig. 1:**
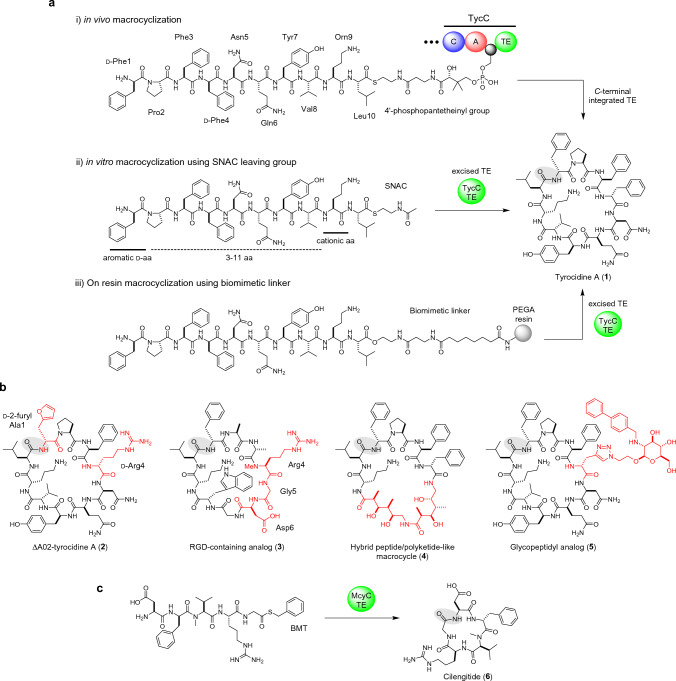
TycC TE-catalyzed macrolactamization. Ring closing sites are highlighted by shading. **a** TycC TE-catalyzed macrolactamization of natural and synthetic substrates. **b** Examples of macrolactams chemoenzymatically synthesized by TycC TE. **c** Synthesis of **6** via McyC TE-catalyzed macrolactamization

Tyrocidine A (**1**) is a head-to-tail cyclized, amphiphilic non-ribosomal decapeptide with potent membrane disruption activity [17]. In 2000, Trauger et al. reconstituted the macrocyclizing step of tyrocidine biosynthesis in vitro, reporting that an overexpressed excised TE domain (TycC TE) involved in tyrocidine biosynthesis cyclized a *seco*-tyrocidine *N*-acetylcysteamine (SNAC) thioester to give **1** [13] (Fig. 1a). This demonstrated for the first time that (i) an excised TE retains macrocyclizing activity in vitro, and (ii) SNAC is an effective low-molecular-weight surrogate of a peptidyl-carrier protein (PCP), a part of the megasynthetase linked at the substrate C-terminus through the phosphopantetheinyl group. This set the stage for detailed analyses of TEs as well as their biocatalytic exploitations. TycC TE requires an aromatic D-aa at the N-terminus and L-Orn at position 9 (Orn9) (Fig. 1a) [13]. The latter was initially thought to play a role as a proton-donor for intramolecular hydrogen-bonding to pre-organize the substrate into a product-like conformation [13]. However, a subsequent study showed that Orn9 can be replaced by non-proton-donating cationic residues like trimethyl-Lys, suggesting that Orn9 may interact with the negatively charged protein surface rather than forming an intramolecular hydrogen bond [18]. TycC TE exhibits remarkable substrate tolerance: an *N*α-amine nucleophile can be substituted with a hydroxy group to achieve macrolactonization [19]. It accepts substrates with various ring sizes ranging from 6 to 14 [20], showing its broad tolerance for substrate length. The catalytic ability of TycC TE is not limited to simple cyclization, but also includes cyclo-dimerization, as it converts a pentapeptide-SNAC to gramicidin S by simultaneously catalyzing ligation and cyclization [13]. Influential work led to the proposal of a minimal cyclization substrate for TycC TE, highlighting the importance of the residue near the ring closure site for enzyme recognition [19].

The biocatalytic potential of TycC TE was then extensively explored [21–28]. TycC TE tolerates oxoester substrates that are tethered to a solid support (PEGA resin) through a biomimetic linker, thus enabling chemoenzymatic synthesis in which combinatorial SPPS (Solid-Phase Peptide Synthesis) chemistry and enzymatic cyclization can be performed seamlessly [21] (Fig. 1a). A library of 192 tyrocidine variants with simultaneous substitutions at positions 1 and 4 was screened for both the minimal concentration of antibacterial activity against *Bacillus subtilis* (MIC) and hemolysis of human erythrocytes (MHC), which led to the identification of a variant (**2**) with an improved therapeutic index (MHC over MIC) [21]. The internal variable region of substrates can accommodate diverse structural motifs (Fig. 1b), such as the RGD sequence to yield an integrin-binding macrocycle (**3**) [24], (*E*)-alkene dipeptide isosteres to give cyclic peptidomimetics [25], fragments of type B streptogramin to produce chimeric macrocyclic antibiotics [26], and polyketide-like building blocks to generate peptide/polyketide-like hybrid molecules (**4**) [27]. Lin et al. incorporated a propargyl glycine residue at position 4, enabling post-cyclization structure diversification via copper(I)-catalyzed alkyne/azide cycloaddition (CuAAC) [28]. By coupling with azido sugars, 247 glycopeptidyl variants of tyrocidine A were synthesized, resulting in the identification of a lipoglycopeptide (**5**) with a sixfold better therapeutic index than natural tyrocidine [28].

Li’s group recently reported that MycC TE in microcystin biosynthesis can cyclize non-native sequences activated by BMT (benzylmercaptan), and utilized it for the chemoenzymatic synthesis of cilengitide (**6**), a head-to-tail cyclic pentapeptide with potent integrin inhibitory activity (Fig. 1c). Substitution of the catalytic Ser with Cys enhanced the catalytic activity, and 250 µM of benzyl mercaptan thioester was cyclized to 49% within an hour of incubation [29].

### TE-catalyzed head-to-side chain macrocyclization

Head-to-side chain cyclization, in which the C-terminal carbonyl is linked to side chain nucleophiles at internal residues to give a “lariat-shaped” product, is one of the structural hallmarks of NRPs. Numerous NRPs with pharmaceutical relevance, such as lipopeptide antibiotics, fall into this subgroup. To date, many TEs that catalyze head-to-side chain macrocyclization have been characterized in vitro. Representatives are TEs involved in the biosynthesis of surfactin [20, 30], calcium-dependent antibiotics (CDA) [31], A54145 [32], daptomycin [32], lichelin [33], fengacin [34], lysobactin [35], polymyxin [36] etc. Compared to the Tyc TE that catalyzes head-to-tail macrocyclization, TEs catalyzing head-to-side chain macrocyclization are often less versatile as biocatalysts, since most exhibit narrow substrate scopes in terms of ring size and residues near ring closing sites. A significant hydrolytic flux of peptide-*O*-TE intermediates is also often reported. Furthermore, some are inactive with a conventional SNAC leaving group and require PCP-loaded substrates [37] or higher activation (i.e., the use of thiophenol as a leaving group) [38]. Ring-opening reverse reactions that hydrolyze the macrocycle products often contribute to low product yields [39]. Nevertheless, the abilities of these TEs to catalyze regio-, stereo-, and chemo-selective cyclizations to yield lariat-shaped products directly from linear substrates provide valuable opportunities to develop efficient chemoenzymatic approaches. Indeed, TEs such as CDA TE and Crp TE were utilized to synthesize variant libraries of daptomycins and cryptophysins, respectively [31, 40]. These TEs are particularly powerful in their abilities to achieve regio-selective macrolactonization, which frequently poses synthetic challenges like low coupling yields and epimerization via chemical methodologies [41].

Early investigations revealed that TE-mediated head-to-side chain cyclization is generally sensitive to alterations in the environment near the nucleophiles. For example, Srf TE in surfactin biosynthesis does not tolerate the stereochemical inversion of the β-OH nucleophile in the acyl chain [20]. Similarly, Syr TE in syringomycin biosynthesis [38] and CDA TE in CDA biosynthesis [42] do not tolerate the inverted Cα-stereochemistry of their nucleophilic residues, L-Ser and L-Thr, respectively. Cyclization is generally chemo-selective, while A15145 TE exceptionally accepts not only the natural nucleophile β-OH L-Thr (macrolactonization) but also β-NH_2_ L-Dap (macrolactamization) [42].

**Fig. 2:**
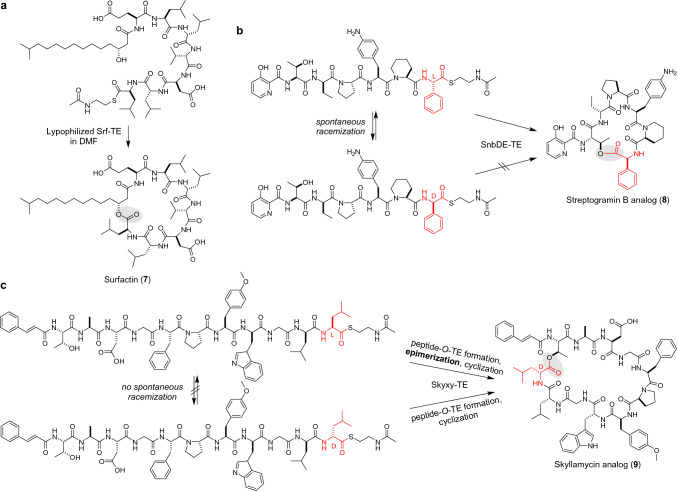
TE-catalyzed head-to-side chain macrocyclizations. Ring closing sites are highlighted by shading. **a** Srf TE-catalyzed macrolactonization in DMF. **b** SnbDE TE-catalyzed stereoselective macrolactonization of a racemization-sensitive thioester. **c** Skyxy TE-catalyzed epimerization/macrolactonization

Substrates with long acyl chains are poorly soluble in the aqueous reaction conditions of TEs, making in vitro characterizations and biocatalytic applications of lipopeptide TEs challenging. Shortening the acyl chains to improve the solubility reportedly compromises the regio-specificity in cyclization [42]. To tackle the solubility issue, Marahiel’s group performed Srf TE cyclization in 100% DMF, which dramatically enhanced the catalytic activity and suppressed hydrolysis to afford surfactin (**7**) in a quantitative conversion (Fig. 2a) [43]. Similarly, the non-polar detergent Brij57 profoundly improved the catalytic efficiency and catalyst lifetime of Tyc TE [18]. These observations highlight the significance of solvent engineering for optimizing the biocatalytic applications of TEs.

The stereoselective nature of TE was combined with dynamic kinetic resolution (DKR) to achieve the stereoselective cyclization of a highly racemizable substrate [44]. The SNAC substrate of SnbDE TE, involved in streptogramin biosynthesis, possesses L-Phg at its C-terminus, which is prone to racemization. However, when starting from a 1:1 racemic mixture of the SNAC substrate, SnbDE TE gave a single stereoisomeric macrolactone (**8**) due to the strict stereospecificity of SnbDE TE for the C-terminal L-Phg. The yield was greater than 50%, due to the in situ stereochemical inversion of the C-terminal D-Phg to L-Phg (Fig. 2b).

Recently, Ma’s group characterized Skyxy-TE in skyllamycin biosynthesis. Skyxy-TE is a bifunctional TE that actively catalyzes the epimerization of a non-racemizable C-terminal L-aa of a linear substrate and performs regioselective macrolactonization to generate skyllamycin analog (**9**) (Fig. 2c) [45]. Epimerization is suggested to occur at the point of peptide-*O*-TE complex formation, and then regioselective macrolactonization affords the epimerized macrolactone. The crystal structure and mutagenesis of Skyxy-TE identified a key Gln residue near the catalytic Ser, and the substitution of this Gln affords racemic products.

**Fig. 3:**
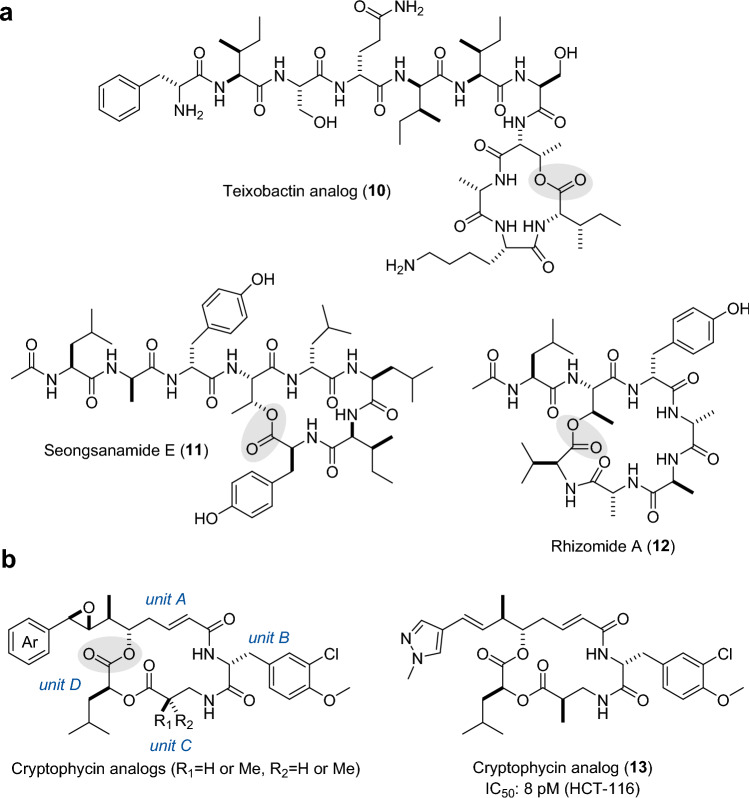
Chemoenzymatically synthesized macrolactones. Ring closing sites are highlighted by shading. **a** Chemical structures of teixobactin analog (**10**), seongsanamide E (**11**), and rhizomide A (**12**). **b** Chemical structures of cryptophycin analogs and a low-picomolar potency analog (**13**)

Teixobactin, a depsipeptide with promising antibiotic activity, is produced by the β-proteobacterium *Eleftheria terrae*, and was isolated using the in situ cultivation method iChip [46]. The teixobactin NRPS possesses duplicated TE domains, sometimes referred to as tandem TEs, and similar architectures are also observed in the NRPSs of lysobactin [35] and arthrofactin [47]. While only the N-terminal TE in the lysobactin system is responsible for macrolactonization, Zhang’s group reported that both TEs can catalyze macrolactonization in the teixobactin system [48]. Although accompanied by substantial amounts of hydrolytic products, the teixobactin TE (Txo TE) afforded teixobactin analogs (**10**) from linear methyl ester substrates, which are much easier to synthesize than conventional SNAC thioesters (Fig. 3a). Txo TE accepted D-Ser as a nucleophile alongside the native D-Thr, but not L-Thr.

Examples of other recent additions include Sgd TE in the biosynthesis of the lipodepsipeptide seongsanamide [39]. Boddy’s group reported that seongsanamide E (**11**), a lariat-shaped peptide cyclized through the β-OH of L-Thr, could not be chemically synthesized from a linear free acid via macrolactonization [39]. However, Sgd TE catalyzed the macrolactonization of a linear SNAC substrate to give seongsanamide E in a 27% yield, without suffering from the epimerization typically occurring in chemical strategies, demonstrating the advantage of enzymatic macrolactonization [39]. Similarly, RzmA TE in the biosynthesis of the lipodepsipeptide rhizomide catalyzes a chemically challenging macrolactonization through the β-OH of L-Thr to give rhizomide A (**12**) from a linear SNAC substrate in a 15% yield, without substantial flux of hydrolysis [49].

Cryptophycin TE (Crp TE) is one of the most successfully applied TEs in the chemoenzymatic synthesis of bioactive macrocycles. The cryptophycins are a class of highly potent microtubule-binding agents isolated from *Nostoc* cyanobacteria [50, 51] and marine sponge [52]. They consist of four unusual building blocks, including phenyl-octenoic acid (unit A), 3-Cl-*O*-methyl-D-Tyr (unit B), methyl β-Ala (unit C), and L-leucic acid (unit D), which are assembled via a PKS/NRPS hybrid system (Fig. 3b) [53]. In the final step of assembly, Crp TE catalyzes the macrolactonization between the δ-hydroxy group in unit A and unit D to construct a 16-membered cyclic depsipeptide scaffold, followed by the stereospecific epoxidation of unit A mediated by CrpE P450 [53]. The biocatalytic potential of Crp TE was demonstrated by Sherman’s group in 2005. Crp TE from *Nostoc* sp. ATCC53789 efficiently catalyzed the macrolactonization of *seco*-cryptophycin-I SNAC thioester with a cyclization: hydrolysis (cyc:hyd) ratio of 10:1 [54]. Crp TE tolerated the variation of the methyl group at β-alaninyl unit C, while it requires an aromatic moiety at unit A [54]. Crp TE was further demonstrated to be tolerant of an activated acylsulfonamide substrate on solid support (PEGA resin), potentially accelerating the preparation of cryptophycin and its analogs [55]. Moreover, Crp TE-mediated macrolactonization was coupled with CrpE P450-mediated stereospecific epoxidation of unit A to generate natural and unnatural cyclic structures with an epoxide moiety, in a one pot manner [53, 56]. Recently, the same group developed a scalable synthesis of linear SNAC substrates with various five- or six-membered heterocycles at unit A and dimethyl derivatives at unit C [40]. Crp TE tolerated heterocycles at unit A and cyclized the SNAC substrates in 69% to 97% conversion, with a cyc:hyd ratio greater than 10:1. Crp TE also cyclized dimethyl derivatives at unit C in 68% to 71% conversions, with a cyc:hyd ratio greater than 7:1. Reactions were conducted on a semi-preparative scale and twelve cyclic products were isolated and tested for their cytotoxicity, which led to the identification of a new derivative (**13**) with single-digit picomolar potency against the HCT-116 human colorectal cancer cell line. Notably, **13** deviated from the requirement of an epoxide group in unit A, which was previously thought to be invaluable for high potency. This study not only provided one of the most potent cryptophycins to date, but also demonstrated the viability of the TE-mediated cyclization strategy in medicinal chemistry [40].

### TE-catalyzed oligomeric macrocyclization

Oligomeric cyclic scaffolds, in which relatively short fragments are oligomerized and cyclized, are another structural hallmark of macrocycles biosynthesized by thio-templated machineries. Ligation and successive cyclization are solely mediated by TEs, which are often referred to as ‘iterative TEs’. The linkages formed by iterative TEs include amide, ester, and thioester, with two to three monomers being oligomerized.

**Fig. 4:**
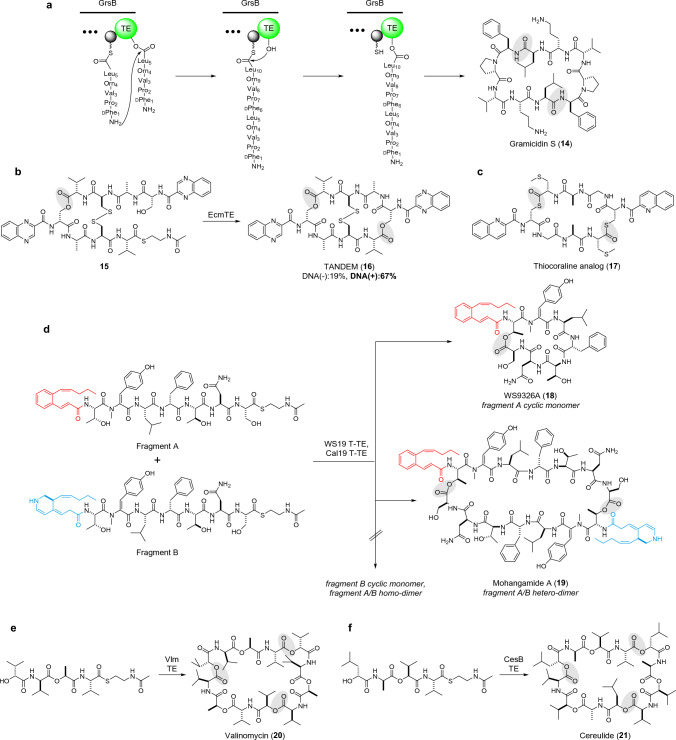
TE-catalyzed cyclo-oligomerization. Amide, ester, and thioester linkages formed by TEs are highlighted by shading. **a** Reverse-transfer mechanism for GrsB TE-mediated cyclodimerization in the biosynthesis of gramicidin S (**14**). **b** Ecm TE-catalyzed macrolactonization. **c** Thiocoraline analog (**17**) chemoenzymatically synthesized by TioS T-TE. **d** WS19/Cal19 T-TE-catalyzed heterodimerization. **e** VlmTE-catalyzed cyclotrimerization. **f** CesB TE-catalyzed cyclotrimerization

The cyclo-oligomerization activity of a TE was initially characterized with EntF TE, involved in enterobactin biosynthesis [57], but later intensively characterized with GrsB TE, which catalyzes dimerization and cyclization of pentapeptidyl precursors to generate gramicidin S (**14**) [58]. Gramicidin S is a head-to-tail cyclic membrane-active decapeptide antibiotic homologous to tyrocidine A [17]. Generation of the decapeptide SNAC from the pentapeptide SNAC substrate indicated the so-called ‘reverse transfer mechanism’ for TE-catalyzed oligomerization, where the peptide-*O*-TE is attacked by a second monomer (SNAC substrate in vitro or PCP-tethered intermediate in vivo) to form a dimerized linear intermediate, then reloaded onto TE to form the peptide-*O*-TE complex [58] (Fig. 4a). GrsB TE tolerates various substrate sizes to catalyze dimerization/trimerization and cyclization to give 6–15 residue macrocycles [58]. The fate of the peptide-*O*-TE (ligation with next monomer or intramolecular cyclization) is thought to be affected by both the pre-folding of the linear intermediate and the catalytic pocket capacity of TE. Notably, TycC TE catalyzes the cyclo-dimerization of a pentapeptide SNAC to generate **14**, indicating that the ligase activity is not confined to a specific subgroup of TEs [13]. Rather, ligation could be a major flux when a peptide-*O*-TE intermediate is too small to fully occupy the catalytic pocket, allowing a second molecule to intrude for ligation. Aligned with this, SurE, a PBP-type TE (see next section for details) that natively accepts an octapeptide substrate, catalyzes ligation/cyclo-dimerization when fed a tetrapeptide substrate [59]. GrsB TE can catalyze cyclo-oligomerization toward mixed monomers to give heteromeric macrocycles, providing an opportunity for biocombinatorial approaches to randomly assemble macrocycles from a pool of small fragments [58].

Oikawa’s group developed a unique strategy for the chemoenzymatic synthesis of quinoxaline/quinoline antibiotics by using the TE in echinomycin biosynthesis (Ecm TE) [60]. This group of compounds binds to double-stranded DNA through bis-intercalation using the quinoxaline/quinoline chromophores, inducing potent antitumor activity. The common cyclic dilactone core is constructed by TEs through cyclo-dimerization of tetrapeptidyl precursors. The SNAC tetrapeptide was successfully converted to triostin A via Ecm TE-mediated cyclo-dimerization followed by disulfide formation, although the efficiency remained low. Ecm TE was further applied to the macrolactonization of an octapeptide SNAC, where the pre-installation of a disulfide bridge in the octapeptide substrate (**15**) substantially improved the product yield. In this way, the synthetic triostin analog TANDEM (**16**) was obtained in 19% yield with a cyc:hyd ratio of 1:2 (Fig. 4b). Notably, a co-incubation with DNA, which sequesters cyclic products, successfully suppressed the product inhibition as well as the undesired hydrolysis of the cyclic product. This approach markedly improved the yield (67%) and cyc:hyd ratio (18:1) of **16** as well as its analogs with DNA-binding activity [60].

A unique thiomacrolactonizing TE in thiocoraline biosynthesis was characterized by Marahiel’s group [61]. An excised TE (TioS T-TE) generated a thiocoraline analog (Fig. 4c, 17) from a tetrapeptide SNAC with a cyc:hyd ratio of 7:1 under optimized conditions. TioS T-TE exhibits tolerance for nucleophiles and accepts D-Ser besides the native D-Cys to accomplish macrolactonization, like Ecm TE.

Yoon’s group recently reported an unusual hetero-dimerizing TE in mohangamide A (**19**) biosynthesis [62]. **19** is a pseudo-dimeric NRP consisting of two nearly identical heptapeptides with different acyl chains (fragments A and B in Fig. 4d). **19** is produced by *Streptomyces* sp. SNM55 along with WS9326A (**18**), the cyclic monomer of fragment A. The WS19 T-TE from SNM55 generated **18** and **19** from mixed SNAC substrates of fragments A and B. Notably, neither the cyclic monomer corresponding to fragment B nor cyclic homodimers of A and B were observed (Fig. 4d), highlighting the unique specificity of WS19 T-TE. The same profile was observed for Cal19 T-TE, a homolog derived from *Streptomyces calvus*, which only biosynthesizes **18** [62]. The mechanism underlying this specificity awaits further investigations.

Trimerizing TEs were characterized in the biosynthesis of the structurally analogous potassium ionophores valinomycin (**20**) and cereulide (**21**). In both cases, the TEs (Vlm TE [63]/Ces TE [64]) were capable of trimerizing tetradepsipeptide SNACs to generate **20** and **21**, respectively (Fig. 4e, f). Ces TE tolerates the valinomycin tetradepsipeptide precursor to give **20** and chimeric macrocycles of **20** and **21**.

### PBP-type TE-catalyzed head-to-tail macrocyclization

**Fig. 5:**
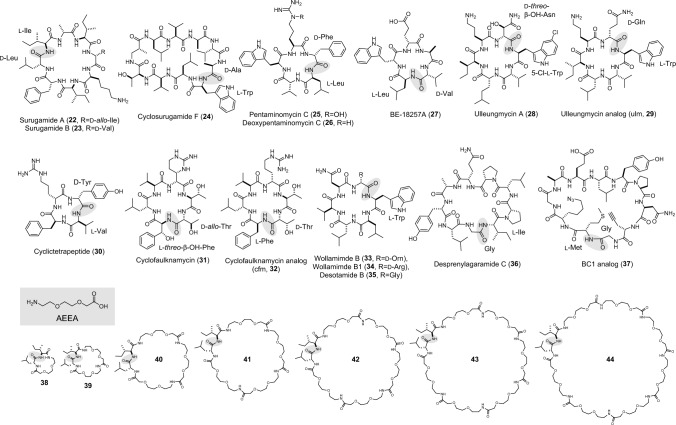
Representative head-to-tail macrolactams that are bio- or chemoenzymatically synthesized by PBP-type TEs, with shading at ring closing sites

Penicillin-binding protein-type thioesterases (PBP-type TEs) are a newly identified group of NRP cyclases. They are composed of two domains: an N-terminal α/β hydrolase domain with a catalytic tetrad (Ser, His, Asn, Lys) and a C-terminal lipocalin-like domain with an eight anti-parallel β-sheet fold. The representative macrocycles that are bio- or chemoenzymatically synthesized by PBP-type TEs are shown in Fig. 5. The chemistry catalyzed by PBP-type TEs is thus far limited to head-to-tail macrolactamization; therefore, they are functionally equivalent to the representative TEs such as TycC TE [13]. However, they are distinct from the conventional type-I TEs in many aspects.

First, PBP-type TEs lack sequence homology to TEs, and thus are evolutionally distinct [65]. They are rather close to the PBP transpeptidases involved in the biosynthesis of cell wall peptide glycan. Second, unlike the TEs that are fused at the C-terminus of NRPS, PBP-type TEs are stand-alone enzymes that act *in trans* to NRPS. The discrete nature allows some PBP-type TEs to act in two NRP assembly-lines to mediate the cyclative release of two distinct peptides [65, 66], in stark contrast to classical TEs that have an exclusive one-to-one relation to their substrates. Therefore, PBP-type TEs inherently possess substantial substrate tolerances. Third, the stereochemical configurations of the residues at the ring-closing site differ between the macrolactamizations catalyzed by PBP-type TEs and those catalyzed by TEs [67]: In vitro characterization of TycC TE, as well as the domain organizations of NRPSs for other head-to-tail cyclic NRPs, revealed that TEs catalyze head-to-tail macrolactamization between an N-terminal D-aa and a C-terminal L-aa. In contrast, PBP-type TEs catalyze head-to-tail macrolactamization between an N-terminal L-aa and a C-terminal D-aa [67]. The requirements of heterochiral residues at the ring closing sites are apparently general among head-to-tail macrolactamizations in NRP biosynthesis, with a small number of exceptions that catalyze homochiral coupling between L-aa [67]. Examples include the TEs involved in the biosynthesis of cyclomarin-type NRPs [68].

Since the first report in 2018 by Wakimoto’s group [65], followed by other groups [69, 70], five PBP-type TEs have been characterized in vitro: SurE in surugamide (**22**–**24**) biosynthesis [65–67, 69, 70], PenA in pentaminomycin (**25**)/BE-18254 (**27**) biosynthesis [59], Ulm16 in ulleungmycin (**28**) biosynthesis [71], FlkO in cyclofaulknamycin (**31**) biosynthesis [72], and WolJ in wollamide (**33**)/desotamide (**35**) biosynthesis [73, 74]. Seminal studies have demonstrated that some PBP-type TEs possess enormous potentials as biocatalysts, while the catalytic efficiencies, scopes of substrate sizes, and preferences of ring-closing residues are diverse among PBP-type TEs. Consequently, a wide array of macrolactams with diverse sequences and sizes has been chemoenzymatically synthesized using PBP-type TEs (Table 1).

**Table 1:** Chemoenzymatically-synthesized macrolactams by using PBP-type TEs

Product	Biocatalyst	Leaving group	Conversion (%)^*a*^	Refs
Surugamide B (**23**)	SurE	SNAC, EG	99	[65, 67, 69, 70, 74]
Cyclosuruamide F (**24**)	SurE	SNAC	–	[66]
Deoxypentaminomycin C (**26**)	PenA, SurE	SNAC, EG	96	[59, 74]
Ulm (**29**)	Ulm16	SNAC, SBMP	–	[71]
Cyclic tetrapeptide (**30**)	Ulm16	SBMP		[71]
Cfm (**32**)	FlkO^*b*^	EG	55^*c*^	[72]
Wollamide B1 (**34**)	WolJ	EG	98	[74]
Desotamide B (**35**)	WolJ	EG	99	[74]
Desprenylagaramide C (**36**)	SurE^G235L^	EG	99^*d*^	[74]
BC1 analog (**37**)	SurE^G235L^	EG	74	[80]
cyc[I-AEEA-DL] (**38**)	SurE	EG	96	[74]
cyc[I-2AEEA-DL] (**39**)	SurE	EG	99	[74]
cyc[I-3AEEA-DL] (**40**)	SurE	EG	99	[74]
cyc[I-4AEEA-DL] (**41**)	SurE	EG	98	[74]
cyc[I-5AEEA-DL] (**42**)	SurE	EG	89	[74]
cyc[I-6AEEA-DL] (**43**)	SurE	EG	66	[74]
cyc[I-7AEEA-DL] (**44**)	SurE	EG	52	[74]

^a^Conversions were accomplished by 5 mol% of PBP-type TEs unless otherwise noted

^b^FlkO was investigated as a form of MBP tag-fused enzyme

^c^Conversion was accomplished by 40 mol% of FlkO

^d^Conversion was accomplished by 10 mol% of SurEG235L

Surugamides consist of two structurally unrelated groups of NRPs: octapeptidyl cyclic surugamides [75–78] (e.g. **22**) and decapeptidyl surugamides (**24** and its linear analog surugamide F) [66, 76, 79]. After each peptide chain is elongated by distinct NRP assembly-lines, both are offloaded by a single stand-alone cyclase, SurE [66]. Consistently, SurE exhibits substantial tolerance for substrate sequence and size: SurE (and its engineered variant [74] described below) efficiently cyclizes peptide sequences that are unrelated to its native substrate, including the sequences of a pentaminomycin analog (**26**) (pentapeptide, 96% conversion) [74], desprenylagaramide C (**36**) (nonapeptide, 99% conversion) [74], and the synthetic peptide BC1 analog (**37**) (undecapeptide, 74% conversion) [80]. Its internal region can accommodate unnatural aa, like those harboring alkyne and azide functionalities. Following the enzymatic cyclization, these clickable residues can be intramolecularly coupled via CuAAC, facilitating the construction of bicyclic scaffolds [80]. The internal region can even be entirely substituted with a non-peptidyl unit, such as 2-[2-[2-(Fmoc-amino)ethoxy]ethoxy]acetic acid (AEEA) [74], indicating that the substrate of SurE does not requires specific structural motifs to facilitate its pre-organization into a product-like conformation. SurE exhibits remarkable tolerance for substrate size, efficiently cyclizing peptides ranging from as few as five aa residues (**38**, one AEEA as an internal residue) to seven to 23 aa residues (**44**, seven AEEAs as internal residues). Although the cyclization of larger substrates is substantially slower, prolonged incubations for up to 12 h resulted in the near-quantitative cyclization of the substrate with seven AEEAs. Notably, no hydrolysis or oligomerization was observed, and monomeric macrocycles were specifically generated for all tested AEEA substrates. SurE tolerates most L-aa at the N-terminus [74, 81], including nonproteinogenic ones like 3,5-dimethyl L-tyrosine [82], but disfavors L-Glu, L-Arg, and L-Pro there [74]. The wild type SurE does not tolerate Gly at the C-terminus, and thus a C-terminal D-aa is obligate for SurE catalysis. It prefers neutral, aliphatic, and aromatic D-aa at the substrate’s C-terminus, while disfavoring anionic/cationic residues at this position [74]. The limitation of SurE regarding the C-terminus can partly be overcome by the use of other wild type enzymes with complementary scopes, such as WolJ, another PBP-type TE in wollamide/desotamide biosynthesis [74]. WolJ is capable of cyclizing sequences with C-terminal Gly, D-Orn, and D-Arg, which are all disfavored as C-terminal residues by SurE [74].

The crystal structure of SurE (*apo*, 6KSU) revealed the basis for its C-terminus specificity: a hydrophobic pocket near the catalytic Ser presumably accommodates the C-terminal D-aa of the peptide-*O*-SurE intermediate [67]. This hypothesis was supported by mutagenesis study [74]: when Gly235 in the pocket was mutated to Ala, the variant lost the activity for the native C-terminal D-Leu sequence but gained a small activity for a C-terminal Gly sequence. The activity for the C-terminal Gly sequence was enhanced as the residue at position 235 got bulkier, and finally, 5 mol% of G235L variant (SurE^G235L^) quantitatively cyclized the C-terminal Gly sequence in a 2 h incubation, showing the correlation between the volume of the pocket and the substrate C-terminal aa. SurE^G235L^, which deviates from the requirements of C-terminal D-aa but obligate for C-terminal Gly, was applied to cyclize D-aa free sequences [74, 80]. A more recent study on FlkO in cyclofaulknamycin biosynthesis revealed its narrower substrate specificity at the C-terminus. FlkO only accepts substrates with small residues such as D-Ala and D-Thr, in addition to the native D-*allo*-Thr [72]. This observation aligns with the occurrence of bulkier residues in the FlkO binding pocket, which significantly reduce the pocket volume [72].

PenA is a PBP-type TE involved in the biosynthesis of two sets of cyclic pentapeptides, pentaminomycins and BE-18254s [83]. Like SurE, PenA is a PBP-type TE acting on two NRPSs [83]. As its product sizes are substantially smaller than those of SurE, PenA was assumed to exhibit a distinct scope regarding the substrate size. Indeed, PenA turned out to be highly specialized for smaller peptides, and accepted a pentapeptide (**26**) (near quantitative conversion) and a tetrapeptide (27% conversion with cyc:hyd ratio of 1:2.7), but not larger substrates [59]. A model structure of PenA revealed an unusually extended loop at its lipocalin domain that covers that substrate binding cleft [59]. Although this loop apparently contributes to narrowing the substrate binding cleft, the basis of its preference for small substrates remains unclear and awaits further investigation.

More recently, Ulm16 in ulleungmycin biosynthesis was biochemically and structurally characterized by Parkinson’s group [71]. Ulm16 tolerates residue substitutions well and, despite its native substrate having a C-terminal D-aa, it efficiently cyclizes a C-terminal Gly sequence, like WolJ and SurE^G235L^. Most notably, Ulm16 is a hyperactive enzyme, which generates **29** from a hexapeptide substrate with butyl 3-mercaptopropionate (SBMP) as a leaving group with a kinetic value of 2.1 × 10^5^ M^−1^s^−1^. This is over 100-fold more efficient than SurE with an octapeptide SNAC substrate. Ulm16 is even more efficient with a tetrapeptide SBMP substrate and cyclized it to give the cyclic tetrapeptide (**30**) with a kinetic value of 3.0 × 10^6^ M^−1^s^−1^, making it the fastest PBP-type TE reported to date. The cyc:hyd ratio for the tetrapeptide substrate is more than 20:1, a substantial improvement compared to that of PenA [59]. Cyclic tetrapeptide scaffolds have remained relatively underexplored due to the synthetic challenges arising from the difficulty in positioning the ring-closing sites within catalytic proximity. As Ulm16 enables facile access to such scaffolds, it can be a valuable tool to explore this chemical space. A structure comparison between SurE and Ulm16 (8FEK) illustrates several notable differences: (i) a shortened loop in Ulm16’s PBP domain and (ii) an inclined Ulm16 lipocalin-like domain relative to the PBP domain, which forms a narrower binding cleft in Ulm16 [71]. These findings set the stage for further investigations to elucidate the protein structure–activity relationships in the PBP-type TEs.

The requirement for nonpeptidic leaving groups is a substantial drawback for biocatalytic applications of NRP cyclases (both TEs/PBP-type TEs). The thioester leaving groups are typically coupled to linear peptides in solution-phase by using coupling regents, which often cause undesired Cα epimerization of the C-terminal residue, necessitating an intensive HPLC purification step to remove the diastereomeric byproducts. This labor-intensive step makes it challenging to synthesize enzyme substrates in parallel or on a larger scale. Alternatively, thioester leaving groups can be installed by using ‘safety-catch’ resin [84], circumventing the solution-phase coupling reaction. However, this necessitates long reaction times for activation and thioesterificaton, and thus the synthesis is still labor intensive and not fully capable of facilitating the production of a large number of enzyme substrates in parallel.

Besides the thioester leaving groups like SNAC and SBMP, PBP-type TEs accept oxo-ester leaving groups, resembling some TEs such as TycC TE and Txo TE. An oxo-ester is less reactive than a thioester, and thus causes reduced catalytic efficiency. However, its compatibility with conventional SPPS chemistry facilitates easier access to enzyme substrates. Seipke’s group tested SurE in an on-resin cyclization strategy [81], a concept similar to the work on TycC TE [22] and GrsB TE [85]. SurE turned out to be compatible with this strategy and cyclized peptides tethered on a solid-support through a biomimetic oxo-ester linkage. Besides the oxo-ester biomimetic linker, which requires five additional synthetic steps for construction on a solid-support, PBP-type TEs tolerate a simpler oxo-ester leaving group, ethylene glycol (EG) [74]. EG can be loaded on Cl-Trt(2-Cl) resin, and then conventional SPPS affords elongated peptides. Cleavage and deprotection of side chains can be performed concomitantly to give peptides functionalized with EG at the C-terminus, thus circumventing the solution-phase coupling step. Although the EG leaving group reduced the kinetic value of SurE by about tenfold compared to the SNAC leaving group, EG-peptides with various sequences and lengths were cyclized by SurE, WolJ, and SurE^G235L^ with good to excellent conversions (Table 1). As this scheme is epimerization-free and virtually omits the need for purifying the linear intermediate, the EG leaving group potentially accelerates the chemoenzymatic parallel synthesis of cyclic peptides using PBP-type TEs. The EG leaving group is also compatible with cyclization mediated by TycC TE [74].

## Conclusion

TEs have been extensively investigated for decades, due to their remarkable potential as macrocyclizing biocatalysts with high levels of chemo-, regio-, and stereoselectivity [86]. TEs potentially facilitate rapid access to diverse macrocycles with various cyclization modes and linkages, directly from non-protected linear precursors. The labor-intensive synthesis of enzyme substrates is one of significant drawbacks for the widespread use of NRP cyclases as chemoenzymatic tools [9]; however, this has been partially addressed by strategies circumventing solution-phase coupling reactions, such as biomimetic on-resin cyclization [21, 55, 81, 85] and modified SPPS using an EG leaving group [74]. These are particularly noteworthy for their compatibility with automated, combinatorial SPPS chemistry, which facilitates rapid access to macrocycles with diverse building blocks beyond canonical amino acids. Other drawbacks of NRP cyclases, including the low stability of excised TEs and the high flux for undesired hydrolytic reactions, may be overcome by recent bioinformatic technologies regarding protein structure prediction, engineering, and design. Broader scope analyses and the construction of panels of TE catalysts with defined scopes may facilitate their practical applications in the synthetic chemistry community. TE-mediated biocatalytic approaches for macrocycles will be further explored in the future, owing to the increasing attention towards cyclic peptides as a therapeutic modality, as well as the growing demand for greener chemistry.
